# Walking in Hypoxia: An Efficient Treatment to Lessen Mechanical Constraints and Improve Health in Obese Individuals?

**DOI:** 10.3389/fphys.2017.00073

**Published:** 2017-02-09

**Authors:** Olivier Girard, Davide Malatesta, Grégoire P. Millet

**Affiliations:** ^1^Athlete Health and Performance Research Center, Aspetar Orthopaedic and Sports Medicine HospitalDoha, Qatar; ^2^Faculty of Biology and Medicine, Institute of Sport Sciences, University of LausanneLausanne, Switzerland

**Keywords:** obesity, altitude training, hypoxic exercise, walking, mechanical loading

Obesity is defined as a body mass index >30 kg/m^2^ and is a major health burden in many parts of the world (Finucane et al., 2011). The incidence of worldwide obesity is escalating at an increasing rate and has more than doubled since 1980. To tackle body fat accumulation and its clinical complications (e.g., diabetes, hypertension, heart disease), aggressive prevention strategies that are mainly based on dieting and lifestyle change have been implemented (Bray et al., 2016). Regular physical exercise such as walking is also generally recommended to increase energy expenditure (Donnelly et al., 2009). Despite an increasing and on-going body of research on cardio-metabolic disorders associated with the obese phenotype (Atkinson, 2014), many questions remain unanswered. Adherence to prescribed or spontaneous exercise remains low in obese patients (Dalle Grave et al., 2011), which raises specific questions on the effectiveness (Malhotra et al., 2015), as well as the objective (Browning and Kram, 2007) and subjective (Annesi, 2000) difficulties of exercising in these patients.

Due to discomfort, it remains unclear how excessive adipose tissue contributes to lower levels of physical activity as well as lower mobility and functional performance. Furthermore, obesity may increase joint stresses during simple locomotion tasks such as walking, which can lead to aberrant mechanics, reduced range of motion in the joints and eventually musculoskeletal pathologies (e.g., lower-extremity osteoarthritis, rheumatoid arthritis and/or low back pain) (Wearing et al., 2006; Browning, 2012; Sheehan and Gormley, 2012). For many obese patients, the reality of musculoskeletal disorders such as knee osteoarthritis may outweigh the eventual benefits of physical activity and weight loss. Altogether, non-compliance by obese patients to current exercise prescriptions and recommendations (Donnelly et al., 2009) suggests that existing exercise regimes do not necessarily meet the needs of this population. Alternative strategies are therefore required and their effectiveness must be clinically validated.

Hypoxic exposure results in any inspired pressure of oxygen under a normoxic value of 150 mmHg (Conkin and Wessel, 2008), while exercising is considered a new therapeutic strategy (Kayser and Verges, 2013; Millet et al., 2016). Until now, the combination of hypoxia and exercise stressors had mainly been investigated in normal weight patients (body mass index <25 kg/m^2^) or lean individuals. The focus of the very few existing studies that included obese patients was on metabolic and body composition changes and not on walking or biomechanical improvement (Netzer et al., 2008; Haufe et al., 2010; Wiesner et al., 2010; Gatterer et al., 2015). Therefore, while cardio-metabolic adaptation was the main target of available studies on obesity (Atkinson, 2014; Verges et al., 2015), biomechanical investigations where hypoxia would be manipulated are also necessary. This is potentially useful to advance basic science as well as to develop effective physical activity recommendations to achieve energy expenditure goals while reducing the risk of musculoskeletal injury in obese patients (Figure 1).

**Figure 1:**
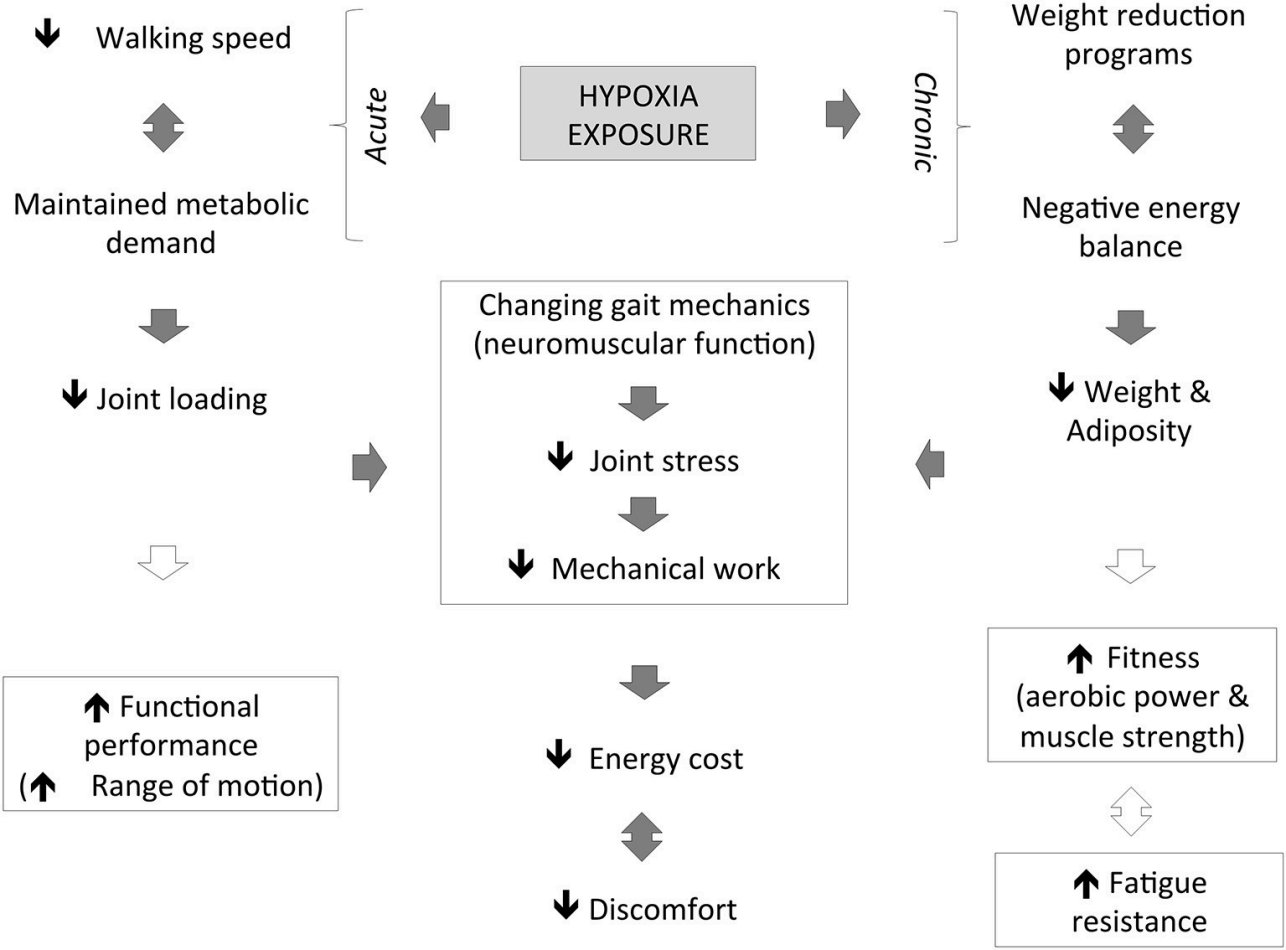
**Illustration of the hypothetical effects of hypoxia exposure on neuro-mechanical constraints associated with walking in obese patients**.

## Locomotion mechanics in obese patients

Compared to lean individuals, preferred walking speeds have been consistently reported to be slower (i.e., typically 10–15%) in obese patients, with walking speeds that also appear inversely related to body mass index (Browning and Kram, 2005; Malatesta et al., 2009). This is presumably because of disproportionately heavier limbs, reduced relative muscle strength and a greater need to maintain balance during ambulation (Sheehan and Gormley, 2012). Obese patients tend to walk with a longer period of double support (i.e., both feet on the ground) and they swing their legs more rapidly (e.g., shorter swing time) with more lateral circumduction (e.g., wider step width) as well as abnormal (e.g., altered distribution) lower-extremity joint loads, absolute ground reaction forces (e.g., excessive peak forces and higher loading rates) and forefoot pressures when compared with non-obese individuals walking at identical speeds (Devita and Hortobágyi, 2003; Wearing et al., 2006; Browning, 2012). To date, there is no clear consensus on the precise nature of mechanical “mal-adaptations” associated with obesity (Sheehan and Gormley, 2012). Hence, whether a more erect sagittal plane posture and greater knee adduction, hip abduction and foot eversion are seen in obese vs. lean individuals remains controversial. As obese patients typically display a large variability in their mechanical stride parameters, it is difficult to identify common characteristics of walking patterns in this population (Wearing et al., 2006). Despite their greater body mass, absolute joint torques at the hip and knee are relatively similar to those of non-obese individuals, while ankle joint torques are approximately twice as large (Wearing et al., 2006). These biomechanical differences require that physical therapists and clinicians should make specific recommendations when prescribing physical exercise to obese patients.

## Weight-perturbation interventions

Increasing the level of physical activity is likely a crucial intervention for an efficient prevention and treatment of obesity (Donnelly et al., 2009). The importance of reducing fat-mass accumulation in obese patients is indisputable; but surprisingly, only a few studies addressing the positive effects of weight loss on changes in walking biomechanics exist. Notwithstanding, massive weight loss produces dramatic reductions in knee forces during walking but when patients walk faster, these favorable reductions become substantially attenuated (Devita et al., 2016).

Because of various interventions (e.g., exercise training, Peyrot et al., 2010) or bariatric surgery (Hortobágyi et al., 2011), mechanical alterations (e.g., lower knee joint loads) and reductions in musculoskeletal pain may minimize the net energy cost. The combination of slower walking speeds and moderate inclines (0.75 m/s, 6°) is another intervention that is metabolically similar to a “normal” level of walking (1.50 m/s, 0°), yet it is associated with reduced net-muscle moments and loading rates (Ehlen et al., 2011). Furthermore, a faster stride frequency when walking speed is held constant (an example of “gait retraining”) likely increases (~5%) energy expenditure without negatively affecting walking mechanics (Russell et al., 2010). Finally, a 10% weight loss through dietary intervention reduced knee compression by 200 N and reduced pain and disability in obese adults with knee osteoarthritis (Messier et al., 2013). Reducing potentially harmful forces in the knee during walking would improve locomotion in obese individuals.

In clinical rehabilitation settings, reducing musculoskeletal loading using lower body-pressure treadmills has an unprecedented popularity. For example, a 2016 meta-analysis indicated that peak and active vertical ground-reaction forces were consistently reduced in artificially weight-reduced healthy individuals; unweighting also provided some horizontal assistance and altered regional loading within the foot toward a forefoot strike (Farina et al., 2017). A potential drawback is that decreased speed with body-weight support will also reduce energy expenditure (e.g., oxygen uptake and heart rate readings) and muscle activation (i.e., with different responses between both stabilizer and propulsive muscles), likely minimizing any stimulus training effect. Use of a lower-body positive-pressure treadmill requires wearing tight neoprene shorts that are then attached to the treadmill and probably limits the range of motion of certain lower extremity joints and balance. One also cannot rule out that an improper arm swing leads to modified gait mechanics.

## Hypoxic exposure

Browning and Kram (2007) calculated that obese patients would need to walk at approximately 1.1 m/s (i.e., close to their preferred walking speed) to have a biomechanically equivalent joint load as lean individuals walking at 1.4 m/s. Consequently, obese patients would have to walk faster than their preferred walking speed to increase exercise intensity and to match the current physical activity guidelines (Donnelly et al., 2009). However, lower-extremity joint loads and the associated risk of musculoskeletal disorders likely increase with walking speed (Browning and Kram, 2007). In this context, acute hypoxia exposure may become advantageous as the mechanical load during physical exercise under hypoxic vs. normoxic conditions would be significantly reduced to achieve the same metabolic effect. In short, hypoxia enabled obese patients to achieve a higher metabolic demand, while a lower walking speed was also likely more protective of the muscles/joints in obese patients with orthopedic comorbidities (Wiesner et al., 2010).

To limit the negative effect of increased level walking speed on mechanical constraints, it is important to determine if walking in an O_2_-deprived environment at a slower speed represents an effective strategy to reduce the load across the lower extremity joints, while providing adequate (i.e., similar to faster walking speeds near sea level) physiological stimulus for weight management. In healthy older community dwellers, changes of time-based gait parameters (e.g., slower cadence, longer stride time, and larger temporal gait variability) from the beginning to the end of a 40-min treadmill walk occurred, but these fatigue-related effects were similar to a 2,600-m simulated altitude or those near sea-level (Drum et al., 2016). Conversely and while exposed to hypoxia, obese subjects may re-organize their neuromuscular function to produce gait patterns that result in different knee joint loading. Based on the findings of studies conducted in healthy populations with lower-body positive-pressure treadmills, we postulated that most muscles that are involved in body-support would demonstrate a reduction in muscle activity as mechanical constraints would decrease with acute hypoxia exposure. Changes in muscle activity are not directly proportional to the changes in “unweighting” for all muscles, and vary widely across muscles [i.e., activation is not decreased in certain muscles (e.g., tibialis anterior, rectus femoris) until considerable unweighting occurs, Sainton et al., 2015, while others (e.g., hip adductor; Hunter et al., 2014) will barely change] and include the severity of hypoxic stress. This type of exercise intervention may be particularly useful to develop familiarity and compliance with regular physical exercise, especially when ambulation becomes less painful (Hootman et al., 2002; Ekkekakis and Lind, 2006).

Hypoxic conditioning consisting of chronic hypoxic exposure or sessions of intermittent exposure to moderate hypoxia repeated over several weeks may induce hematological, vascular, metabolic and neurological effects (Verges et al., 2015). This is presently considered as a promising therapeutic modality for several pathological states (e.g., heart failure, stroke, spinal cord injury patients) including obesity (Urdampilleta et al., 2012; Kayser and Verges, 2013; Millet et al., 2016). Continuous hypoxic training (low intensity endurance exercise for 90 min at 60% of the heart rate at maximum aerobic capacity, three times per week for 8 weeks; inspired fraction of O_2_ = 15%) in overweight subjects (body mass index >27 kg/m^2^) leads to larger (+1.14 vs. 0.03 kg) weight loss than similar training in normoxic environments (Netzer et al., 2008). The effectiveness of such a low-intensity intervention remains questionable over a longer period of 8 months since Gatterer et al. (2015) did not report higher reductions in body weight between hypoxic and normoxic interventions. However, the biomechanical consequences of chronic hypoxic interventions on the walking pattern of obese patients are simply unknown.

To date, epidemiological reports associate the moderate altitude of residence to lower obesity prevalence without clear underlying mechanisms (Voss et al., 2014; Woolcott et al., 2016). Recently, it was reported that O_2_ variations in organic systems may lead to considerable (3%) weight loss (yet of undefined composition) and improve metabolic and cardiorespiratory health (Netzer et al., 2008; Kayser and Verges, 2013; Kong et al., 2014). This leads to the suggestion that sustained hypoxia may be of benefit to weight management programs in obese patients (Millet et al., 2016). For prolonged exposure, the so-called “altitude anorexia” mechanisms that lead to a reduced appetite in altitude cannot be ruled out (Tschop and Morrison, 2001). The explanation for this phenomenon remains unclear but has been related to a modification in appetite regulation hormones (Shukla et al., 2005).

## Perspectives

### Acute hypoxia

- On a lower-body positive-pressure treadmill, faster speeds are required to reach similar exercise intensities than on a normal treadmill (Farina et al., 2017). Reportedly, faster walking and running speeds (6–16 km/h) rather than increases in percent body weight (50–100%) cause a greater maximum plantar force on a lower-body positive-pressure treadmill (Thomson et al., 2017). These observations are limited in scope to healthy male runners. When combined, acute hypoxia exposure (normobaric hypoxia) to artificially increase the metabolic load and exercise on body positive-pressure or aquatic treadmills to decrease the mechanical strain might be clinically relevant. Deeper investigations of obese patients walking at slower speeds (e.g., ranging 0.5–3.5 km/h) with and without hypoxic exposure are required and are likely to alter the relationship between speed and muscle unweighting on mechanical strain as reported in similar studies (Thomson et al., 2017). While exercising at simulated altitudes ranging from 2,500 to 3,500 m is commonly implemented (Haufe et al., 2010; Kong et al., 2014; Gatterer et al., 2015), the optimal degree of hypoxic severity (i.e., maximize physiological adaptations with limited negative consequences) is unknown (e.g., Urdampilleta et al., 2012 for intermittent hypoxic exercise recommendations), and requires further study. However, it is likely that due to the maladaptive side effects associated with hypoxia (i.e., sleep apnea, intermittent hypoxemia), obese patients would not tolerate a high-altitude (Dempsey and Morgan, 2015). For safety reasons, we speculate that hypoxic training could be performed at a simulated altitude lower than 3,500 m.
- Thus far, the available obesity-related studies investigated gait mechanics prior to any signs of fatigue. Perturbations in muscle force generation could reduce the ability of fatigued muscle groups to attenuate to ground reaction impact forces, thereby exaggerating gait abnormalities, and likely increasing the fall risks during locomotor tasks (Himes and Reynolds, 2012). To date, there is a dearth of information pertaining to the changes in gait and balance control over time as obese patients start to fatigue. It is also unknown whether there is a cumulative effect of hypoxic exposure and exercise-induced fatigue on locomotion mechanics that may increase fall risks in obese individuals who are exercising for a prolonged time at moderate altitude.
- Walking includes an inherent fall risk based only on the mere exposure to gradients and surface variations (e.g., stair climbing, rocky paths) that would influence the load applied to the weight-bearing joints of obese individuals. Surface electromyography (EMG) recording during various locomotive tasks would elucidate muscle activation strategies used by obese patients to cope with these situations, including whether hypoxia exposure modifies neuromuscular responses. While the surface EMG may be feasible in severely obese individuals (Minetto et al., 2013), decomposition of surface EMG signals into time-frequency components, wavelet components and degrees-of-freedom force functions may provide further insight into the contributions from the neuromuscular system (Hamid Nawab et al., 2010).

### Chronic hypoxia

- Weight loss is an important method for the treatment of obesity and its associated comorbidities. It is possible to measure the net energy cost of level-walking in obese patients by investigating the effect of decreased body mass on gait pattern and external mechanical work (i.e., simple inverted pendulum modeling to approximate the energy required to raise and accelerate the center of mass; Malatesta et al., 2013). To date, moderate-intensity continuous training is the type of physical activity most frequently recommended to obese patients (Donnelly et al., 2009). That said, growing evidence suggests that high-intensity interval training is a time-efficient approach in this population (Kong et al., 2016). However, this training was rated as less pleasant and less enjoyable than an isocaloric session of moderate-intensity continuous exercise (Decker and Ekkekakis, 2017). Future studies are warranted that compare the effects of various exercise modalities/intensities in addition to hypoxia exposure on the changes in gait pattern.
- Hypoxic training embraces different methods as “live high–train high,” “live high–train low,” or “live high–train low” interspersed with hypoxic training for additional sessions (“live high–train low and high”) that can be conducted in normobaric or hypobaric (natural) conditions with the use of artificial devices or by ascending to elevated terrestrial environments (Millet et al., 2013). To our knowledge, there are no studies that compared the influence of these different altitude-training methods on the weight loss and gait mechanics in obese patients. Consequently, how different physiological adaptations and different degrees of body mass loss associated with various hypoxic training methods would specifically affect gait pattern in obese patients remains undetermined.
- Walking and hiking for hours at low-to-moderate intensity in mountainous areas is a popular and safe outdoor activity, even for obese patients (Neumayr et al., 2014), and should therefore be recommended to increase physical activity in this population. The potential identification of a significant difference between simulated (i.e., normobaric hypoxia) and terrestrial (i.e., hypobaric hypoxia) altitudes is also clinically relevant. Interestingly, Degache et al. (2012) reported that real altitude superiorly decreased postural stability. Investigations that compare normobaric to hypobaric hypoxia in obese patients are currently lacking.

## Conclusion

Obese patients should be enthusiastically encouraged to engage in regular physical activity for the improvement of cardio-metabolic health, with exercises selected that minimize the joint load and pain as much as possible. Walking at slower speeds under hypoxic conditions would reduce joint loading (and the risk of musculoskeletal injury/pathology), while ensuring an adequate exercise stimulus for weight management. Alternatively, hypoxic conditioning may be an appropriate form of exercise training for obese patients as it can lead to effective weight loss due to a negative energy balance. These acute and chronic hypoxic-related interventions would contribute to the support of an appropriate and individualized prescription for obese patients to reduce the biomechanical load involved in walking and eventually improve training adherence (Figure 1).

## Author contributions

All authors listed, have made substantial, direct and intellectual contribution to the work, and approved it for publication.

### Conflict of interest statement

The authors declare that the research was conducted in the absence of any commercial or financial relationships that could be construed as a potential conflict of interest.
